# Development and Function of Secondary and Tertiary Lymphoid Organs in the Small Intestine and the Colon

**DOI:** 10.3389/fimmu.2016.00342

**Published:** 2016-09-06

**Authors:** Manuela Buettner, Matthias Lochner

**Affiliations:** ^1^Central Animal Facility, Institute of Laboratory Animal Science, Hannover Medical School, Hannover, Germany; ^2^Institute of Infection Immunology, TWINCORE, Centre for Experimental and Clinical Infection Research, a joint venture between the Medical School Hannover (MHH) and the Helmholtz Centre for Infection Research (HZI), Hannover, Germany

**Keywords:** isolated lymphoid follicles, cryptopatch, lymphoid tissue inducer cells, tertiary lymphoid organs, small intestine, large intestine

## Abstract

The immune system of the gut has evolved a number of specific lymphoid structures that contribute to homeostasis in the face of microbial colonization and food-derived antigenic challenge. These lymphoid organs encompass Peyer’s patches (PP) in the small intestine and their colonic counterparts that develop in a programed fashion before birth. In addition, the gut harbors a network of lymphoid tissues that is commonly designated as solitary intestinal lymphoid tissues (SILT). In contrast to PP, SILT develop strictly after birth and consist of a dynamic continuum of structures ranging from small cryptopatches (CP) to large, mature isolated lymphoid follicles (ILF). Although the development of PP and SILT follow similar principles, such as an early clustering of lymphoid tissue inducer (LTi) cells and the requirement for lymphotoxin beta (LTβ) receptor-mediated signaling, the formation of CP and their further maturation into ILF is associated with additional intrinsic and environmental signals. Moreover, recent data also indicate that specific differences exist in the regulation of ILF formation between the small intestine and the colon. Importantly, intestinal inflammation in both mice and humans is associated with a strong expansion of the lymphoid network in the gut. Recent experiments in mice suggest that these structures, although they resemble large, mature ILF in appearance, may represent *de novo*-induced tertiary lymphoid organs (TLO). While, so far, it is not clear whether intestinal TLO contribute to the exacerbation of inflammatory pathology, it has been shown that ILF provide the critical microenvironment necessary for the induction of an effective host response upon infection with enteric bacterial pathogens. Regarding the importance of ILF for intestinal immunity, interfering with the development and maturation of these lymphoid tissues may offer novel means for manipulating the immune response during intestinal infection or inflammation.

## Introduction

Lymphatic organs were described very early in history. The first description of the spleen was made in Egypt 3000 years B.C., and the mesenteric lymph node was mentioned by Herophilus in 335–280 B.C. (1, 2). The thymus was characterized in the fifteenth century, but the immunological function of these organs was recognized not until the twentieth century (2, 3). The thymus and bone marrow are referred to as primary lymphatic organs and represent the places in the body where T and B lymphocytes development and selection takes place. Secondary lymphatic organs (SLO), such as the spleen and lymph nodes (LN), develop at predetermined locations during embryonic development and provide the microenvironment that is required for lymphocyte activation and differentiation into regulatory or effector cells. The strategic positioning of SLO allows optimal sampling of self- and non-self-derived antigens that reach the SLO *via* the bloodstream (spleen) or the afferent lymphatics, which also transport antigen-presenting cells from tissues toward the SLO. In addition to the programed development of SLO, it is now well appreciated that structured lymphoid organs can also develop after birth in response to an ongoing immune reaction. These organized lymphoid aggregates resemble SLO and are designated as tertiary lymphoid organs (TLO) or ectopic lymphoid tissues. Formation of TLO has been observed in almost every tissue of the body under inflammatory conditions associated with autoimmunity, infection, or cancer [summarized in Pitzalis et al. (4)].

The mucosal surfaces of the body represent a major entry site for non-self antigens. It is, thus, not surprising that these barrier sites are surveyed by specific SLO that can be differentiated according to their location as nasal-associated lymphoid tissues (NALT), bronchus-associated lymphoid tissues (BALT), or gut-associated lymphoid tissues (GALT). It is, in particular, the intestine that has evolved a range of unique lymphoid organs, reflecting the extraordinary challenge for the intestinal immune system of maintaining tolerance to food antigens and the complex commensal microbiota, while at the same time preserving tissue integrity and the ability to fight harmful pathogens. In this review, we will focus on the different types of intestinal lymphoid organs, their development, and function in intestinal inflammation and infection. We will, furthermore, point out similarities and specific differences regarding the development and maturation of lymphoid tissues between the small intestine and the colon. A special focus will be set on isolated lymphoid follicles (ILF), which show features of TLO such as the postnatal development and the requirement for additional activating signals to promote their formation.

## Programed SLO Development in the GUT

The principal molecular mechanisms that govern the development of SLO have been extensively characterized during the past two decades (5, 6), and various cell types and factors were identified to be crucial for their development. In the gut, a chain of several LN embedded in the mesenteric membranes drain antigens from the intestine. Despite their common location, the mesenteric (m)LN that drain the small intestine and colon in mice can be separated anatomically and by distinct specificities in their immunological functions (7). The development of mLN follows the general molecular processes described for LN formation, which we will first summarize here, before pointing out major similarities and differences to the development of another major SLO in the gut, the Peyer’s patches (PP).

Similar to all other LN, mLN formation starts during embryogenesis *in utero* and proceeds until the development is completely finished after birth. The mLN are the first LN to develop in the mouse, probably starting already around embryonic day (E) 9–12.5 (8). Nerve fibers were identified as potential producers of retinaldehyde dehydrogenase 2 (RALDH2) (5, 9), an enzyme that converts retinal into retinoic acid (RA). RA induces the production of the chemokine CXCL13 by mesenchymal cells in the LN primordium (also designated as LN anlagen). The origin of these mesenchymal cells is not completely clear, but adipocyte progenitor cells were shown to differentiate into LN stromal cells *via* lymphotoxin beta receptor (LTβR) signaling and upregulation of CXCL13 (10). At E13, mesenchymal cells surround endothelial cells (EC), which express EC markers such as podoplanin (also known as gp38), intercellular adhesion molecule (ICAM)-1, and Lyve1^+^ and produce CCL21 (5, 11). CXCL13 and CCL21 together attract a population of CD3^−^ CD4^+/−^ CD45^+^ lymphoid tissue inducer (LTi) cells into the developing LN primordium (5, 11). LTi cells differentiate from fetal liver cells and have been characterized as IL-7Rα^+^, RA receptor-related orphan receptor (ROR)γt^+^, CXCR5^+^, CD117^+^, and RANKL^+^ (12). It has become clear that LTi cells belong to a group of lymphoid cells that is now commonly designated as innate lymphoid cells (ILC) (13). LTi cells resemble group 3 ILC, due to the shared requirement for RORγt expression for their generation and function. Yet, LTi cells may form an independent ILC subset, since they were shown to develop from a specific LTi precursor aside from all other ILC subsets (14, 15). Importantly, LTi cells also express lymphotoxin (LT) α_1_β_2_ on their surface, which binds to LTβR on gp38^+^ stromal lymphoid tissue organizer (LTo) cells (11). Notably, stromal LTo cells express markers found on mesenchymal cells such as platelet-derived growth factor receptor (PDGFR) α (11), indicating that these cells originate from the mesenchymal cells present in the LN anlagen. The binding of LTα_1_β_2_ and LTβR is essential for LN development, as deficiency for LTα, LTβ, or LTβR results in the absence of LN (8, 16, 17). Therefore, the initial clustering of LTi cells and their interaction with stromal LTo cells marks an important step in LN development. Activated LTo cells start to express high levels of ICAM-1, VCAM-1, and MAdCAM-1 (11), which paves the way for further recruitment and retention of hematopoietic cells into the developing LN. IL-7 and RANKL (also known as TRANCE) produced by LTi and LTo cells were both found to be inducers of LTα_1_β_2_. Likewise, LTα_1_β_2_ signaling to LTβR leads to the production of IL-7 and RANKL, resulting in a positive feedback loop, and the accumulation of more LTi cells to the LN primordium (18). Loss of IL-7 or RANKL was shown to result in abnormal LN microarchitecture or even complete absence of LN due to altered LTi cell migration or high endothelial venule (HEV) formation, respectively (18, 19). The connection of HEV and lymphatic vessels to the LN mediated by LTo cells *via* VEGF-C production enables lymphocytes to colonize the LN and to migrate to their distinct B- and T-cell zones (20). LTo cells eventually differentiate into LN stromal cell subsets such as fibroblastic reticular cells (FRC) and follicular dendritic cells (FDC).

## Peyer’s Patches Development

The development of PP starts on E12.5–15.5 (21, 22). Similar to LN development, LTi cells play a pivotal role in the formation of PP, as in the absence of LTi cells in RORγt- or ID2-deficient mice PP fail to develop (23, 24). Recruitment of LTi cells to the PP primordium depends on the chemokine CXCL13 and its receptor CXCR5. However, whereas PP were described to be absent or strongly reduced in CXCL13- or CXCR5-deficient animals (25, 26), mLN were still present, even in mice with a combined deficiency in CXCR5 and CCR7, the receptor for CCL19/CCL21 (26–28). The expression of the adhesion molecule β1 integrin on LTi cells directs them to VCAM1^+^ stromal LTo cells (29), and IL-7/RANKL expression by LTo cells leads to an upregulated expression of LTα_1_β_2_ by LTi cells (30). In contrast to LN development, however, both IL-7 and RANKL seem not to be pivotal for the development of PP, as PP primordia were detectable in IL-7^−/−^ mice at E18 (31) and small PP develop in RANKL^−/−^ mice (32). More important in the formation of PP seems to be the IL-7Rα or receptor tyrosine kinase Kit expression on LTi cells (19, 21, 31). The formation of PP primordia in IL-7Rα^−/−^ mice is disturbed and not detectable, and a defect Kit/Kit ligand axis resulted in a reduced PP development (19, 21). Importantly, the first cell population unique for PP formation and responsible for the clustering of LTi cells in the intestine is of hematopoietic origin. These cells were detected to be CD45^+^, IL7Rα^−^ CD4^+^, CD3^−^ CD11c^+^ and showed similarities to dendritic cells (DC) from the adult spleen (22). These CD11c^+^ cells express two molecules known to be pivotal in PP formation: LTβ and the receptor tyrosine kinase RET. Deficiency for one of these molecules resulted in complete loss of PP development. Furthermore, RET signaling was observed to be dependent on different RET ligands (Artn and Nrtn) expressed on VCAM1^+^ stromal cells. However, the loss of one of these ligands only resulted in a reduced number of PP. Thus, although similar molecules and signaling pathways govern both the formation of PP and LN, there are specific differences in the relative requirement for specific cell types and factors during their development.

## Special Lymphoid Organs of the Small Intestine – The CP/ILF Network

**Figure 1:**
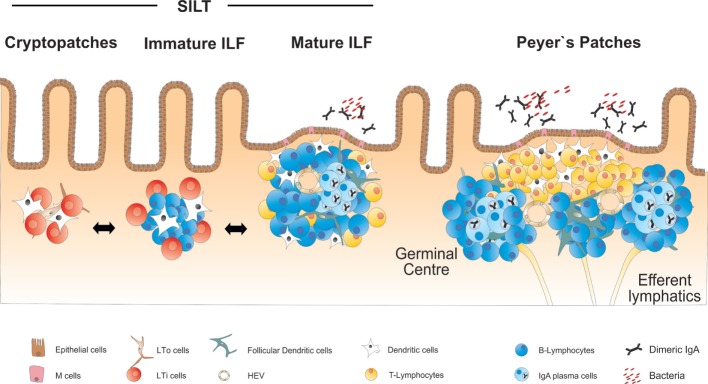
**Overview on the anatomy and structure of CP, ILF, and PP in the small intestine**. SILT consists of a dynamic continuum of structures ranging from small cryptopatches (CP) to large mature isolated lymphoid follicles (ILF). CP start to develop into immature ILF by recruiting B cells. Mature ILF contain one big B cell follicle and develop germinal centers, vascular structures, and a follicle-associated epithelium. PP represent the most structured lymphoid organs in the intestine, containing several B cell follicles and distinct T and B cell areas.

In addition to the PP, the small intestine harbors a large number of organized lymphoid structures that are commonly designated as solitary intestinal lymphoid tissues (SILT). In contrast to PP and all other secondary lymphoid organs, SILT development in the small intestine of mice is initiated during the early postnatal phase and starts with the accumulation of RORγt^+^ IL-7Rα^+^ LTi cells into small clusters named cryptopatches (CP), according to their anatomical location at the bottom of intestinal crypts (33, 34). While in the first days after birth lymphocytes in the intestine are almost exclusively found in the nascent PP (35), CP start to recruit lymphocytes during the following weeks and develop into ILF. Around 100–200 ILF can be found in the small intestine of a mouse, that contain variable numbers of cells and can reach the size of a single PP follicle (36). Large, mature ILF contain mainly B cells as well as a number of interspersed LTi cells and few CD4^+^ T cells, which do, however, not segregate into a confined T cell zone (34, 36). ILF and their CP precursors also contain CD11c^+^ CD11b^−^ DC, which play an important role for the development and structural maintenance of ILFs (37). Most of the B cells within ILF are conventional B-2 B cells, of which around 10% represent IgA^+^ plasma cells (36). Accordingly, mature ILF have been shown to contain germinal centers and serve as inductive sites for antigen-specific IgA responses (38). In addition, ILF have been proposed to be a major site for T cell-independent B cell class switch to IgA (39). Similar to PP, the epithelium adjacent to mature ILF shows the characteristics of a follicle-associated epithelium containing M cells that allow for direct uptake of antigens from the intestinal lumen (36, 40). An overview on the structure and cellularity of the different lymphoid organs of the small intestine is given in Figure 1.

The development of ILF follows similar molecular principles that have been described for the development of PP, such as the interaction of LTα_1_β_2_ expressed by LTi cells with the LTβR on VCAM-1^+^ stromal LTo cells as a critical requirement for the development of CP (40, 41). Transition from CP into ILF in the small intestine depends on the secretion of the B cell recruiting chemokine CXCL13 by stromal cells and CD11c^+^ DC within CP/ILF (37). Consequently, either the deficiency for CXCL13 or its receptor CXCR5 results in the absence of ILF, whereas the numbers of CP is not affected (37, 42). It should be emphasized here that the development of CP, although initiated after birth, proceeds in the absence of any exogenous inflammatory stimuli. Furthermore, the formation of small intestinal SILT strictly depends on LTi cells, as evidenced by the absence of these structures in RORγt-deficient mice, which lack all LTi cells in the intestine (34, 39). Taking this into account, it is, thus, reasonable to consider ILF as true SLO. Unlike the development of all other SLO, however, the formation of ILF is not completely “programed,” and still requires additional input from environmental factors.

Evidence for the requirement of specific dietary products for the development of SILT stems from studies showing that mice deficient for the Aryl hydrocarbon receptor (Ahr) show an almost complete lack of CP/ILF in their small intestines, while the formation of PP or LN was not affected (43, 44). Natural ligands for the Ahr include polyphenols and glucosinolates that can be found in Brassicaceae plants (such as broccoli or cauliflower). Feeding mice with a synthetic diet devoid of any plant-derived products resulted in the absence of CP and ILF at 4 weeks after birth, a phenotype that could be completely restored by the addition of the Ahr ligand indole-3-carbinol to the diet (43). Importantly, tissue-specific deletion of Ahr in RORγt^+^ ILC was sufficient to impair the development of CP, further demonstrating the importance of these cells for CP/ILF induction. Although the exact molecular mechanisms remain elusive, it is likely that Ahr does not regulate the function, but rather the pool size of RORγt^+^ ILC after birth, since ILC-specific loss of Ahr was shown to interfere with the postnatal expansion/survival of these cells (43, 44). Vitamin A, a dietary component, was also recently found to be required for normal numbers of RORγt^+^ ILC and ILF formation (45). Although the different stages of the SILT (CP, immature and mature ILF) were present in mice fed with a vitamin A-deficient diet, their total numbers were reduced in the middle and distal part of the intestine. It is likely that the development of fewer SILT in these mice is a consequence of the reduced numbers of RORγt^+^ ILC upon vitamin A deficiency.

Besides this unexpected role of dietary products in the initial steps of ILF formation, it is less surprising that the intestinal microbiota also plays a role in the regulation of SILT development. In this regard, it has been observed that the small intestine of germ-free mice contain normal numbers of CP and some small immature ILF which harbor only few B cells, whereas the transition into mature ILF depends on the presence of a bacterial microbiota (33, 46, 47). A range of receptors and adaptor molecules involved in the recognition of bacteria-derived molecular patterns, including toll-like receptors (TLR) 2/4, myeloid differentiation primary response gene (MyD) 88, and nucleotide-binding oligomerization domain-containing protein (NOD) 2, were shown to contribute to the maturation of small intestinal ILF (47). In the same study, a more specific role in the early transition of CP into immature ILF was proposed for NOD1, which recognizes peptidoglycans (PGN) derived from Gram-negative bacteria. NOD1, which is expressed in small intestinal epithelial cells, induces upon recognition of PGN the secretion of factors, such as CCL20 or β-defensin 3 (mBD3). Both factors are ligands for CCR6, which is expressed by B cells and LTi cells. Thus, activation of NOD1 may regulate the formation of ILF by activating LTi cells and by supporting the CCR6-dependent recruitment of B cells to CP (48). Supporting this hypothesis, mice deficient either for CCR6 or mBD3 fail to develop ILF, and a similar phenotype could be observed in mice treated with a CCL20 neutralizing antibody (47).

## Lymphoid Organs of the Colon – Similarities and Differences to Small Intestine

**Figure 2:**
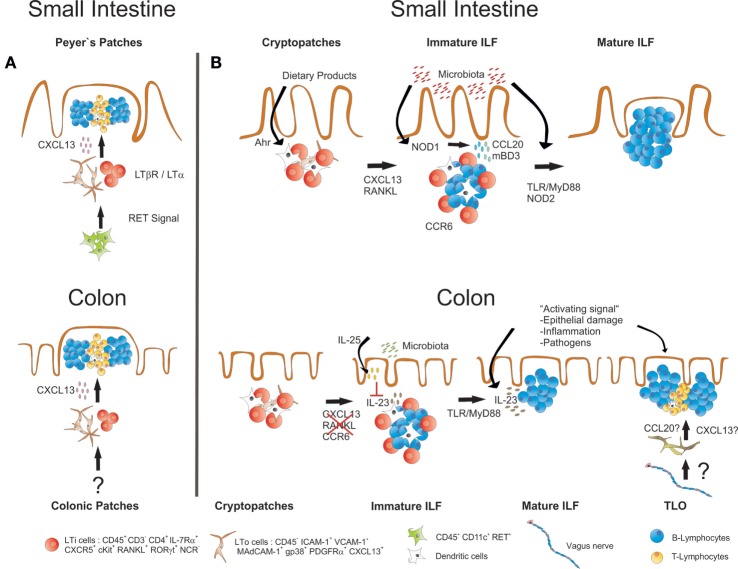
**Similarities and differences in the development of small intestinal and colonic lymphoid tissues**. **(A)** Development of both PP and colonic patches starts before birth with the clustering of LTα_1_β_2_-expressing IL-7Rα^+^RORγt^+^ LTi cells together with LTo cells. LTi cell clustering and further development of both PP and colonic patches requires expression of CXCL13 and is dependent on LT-signaling. Whether the initial clustering of LTi cells in the colon depends on RET-expressing IL7Rα^−^CD11c^+^ cells, as it has been described for PP development, is not known. **(B)** ILF formation in both small intestine and colon starts after birth with the clustering of IL-7Rα^+^RORγt^+^ LTi cells in CP. Maintenance and further development of these structures into ILF requires LT signaling in both small intestine and the colon. In the small intestine, also signaling *via* the Ahr and the expression of CXCL13, RANKL and CCR6 is required, and the commensal microbiota induce ILF formation and maturation *via* activation of signaling pathways that include NOD1, NOD2, TLRs, and MyD88. In the colon, ILF formation does not seem to critically depend on CXCL13, RANKL, and CCR6. There, the presence of commensal microbiota rather inhibits ILF maturation, probably by inducing IL-25 production from the epithelium, which in turn diminishes the secretion of IL-23, a cytokine that specifically promotes ILF maturation in the colon. Signals associated with intestinal inflammation or infection with enteric pathogens also induce ILF formation and maturation. Intestinal inflammation or infection may also result in the induction of TLO that form independently of LTi cells. TLO may be discriminated from ILF by structural differences such as the presence of T cell areas. A role for the intestinal nervous system for TLO induction in intestinal inflammation has been suggested.

Interestingly, most of the factors and molecular mechanisms that govern the development of PP and SILT have been analyzed in the small intestine, while the formation of the respective lymphoid tissues in the colon has not been comprehensively studied until recently. Similar to the small intestine, also the colon of mice harbors distinct lymphoid tissues. Colonic patches represent the equivalents of PP in the small intestine. They are usually composed of two or more large B cell follicles with separate T cell areas and contain CD35^+^ FDCs as well as vessels with HEV. As in the small intestine, also SILT exists, which resemble in its appearance its small intestinal counterpart, ranging from small CP-like structures to mature colonic ILF containing one big B cell follicle with CD35^+^ FDC, but no defined T cell area (49, 50). As for PP, the development of colonic patches starts *in utero* with clustering of RORγt-expressing LTi cells and their interaction with VCAM-1^+^ stromal cells. Although the initial clustering of LTi cells at colonic patch primordium seems to be independent of LTα_1_β_2_–LTβR interaction, the further development into colonic patches strictly requires activation of this pathway as well as the sustained expression of CXCL13 (49). SILT development in the colon starts after birth with the clustering of LTi cells into CP-like structures surrounded by CD11c^+^ cells, and the successful formation of these structures depends again on signaling *via* the LTβR pathway. Despite these important similarities, it is getting increasingly clear that also striking differences exist between the processes of lymphoid tissue formation in the small intestine and the colon (summarized in Figure 2). CXCL13 for example, while required for the formation of colonic patches and the recruitment of B cells into small intestinal CP, seems to be dispensable for colonic SILT formation, as numerous colonic ILF were observed in CXCL13-deficient mice (49). Likewise, ILF formation is blocked in the small intestine of mice deficient for RANKL, presumably due to a role for this cytokine in inducing CXCL13 expression in stromal cells. In contrast, ILF formation is not affected in the colon of RANKL-deficient mice (51). Furthermore, colonic ILF develop normally in CCR6-deficient mice, even though colonic LTi cells express CCR6, and expression of the ligand CCL20 was shown to be decreased in the colon of LTα-deficient mice, which fail to develop colonic lymphoid tissues (49). This indicates that in contrast to the small intestine, SILT formation in the colon is less dependent on the CCR6–CCL20 axis. Most striking, however, is the notion that microbiota-derived signals seem to be much less important for the formation of mature colonic ILF as compared to the small intestine. Two recent studies showed that in germ-free mice the formation of the whole spectrum of SILT in the colon is not significantly impaired (49, 50). Yet, MyD88 signaling was found to be necessary for full colonic ILF maturation, even in the absence of the microbiota (49). Whether MyD88 signaling is induced by activation of the IL-1R family, which also signal *via* the MyD88 adaptor, or the release of endogenous TLR ligands is, however, not clear so far. It should be mentioned in that respect, that in an earlier study a role for TLR2/4 was shown for colonic ILF maturation, however in a microbiota-dependent manner (47). Nevertheless, Donaldson and colleagues even found the numbers of colonic ILF increased in germ-free mice, which could be corrected in their study by transferring these mice to a conventional housing (50). Interestingly, intestinal colonization of germ-free mice with microbiota was accompanied by upregulation of colonic IL-25 expression. IL-25 is produced upon microbial colonization by the intestinal epithelium, and it has been demonstrated that commensal microbiota-induced IL-25 production dampens the activity of RORγt^+^ ILC and reduces IL-23 expression in the intestine (52, 53). Indeed, colonic ILF development was increased in IL-25-deficient mice (50). This was associated with enhanced expression of IL-23, suggesting that IL-25 may influence colonic ILF maturation by negatively regulating IL-23 production. The authors could confirm this hypothesis by showing that IL-23p19-deficient mice displayed a colon-specific decrease in ILF numbers, and identified colonic (but not small intestinal) CD11c^+^ cells within ILF as a source for IL-23 production. Thus, the commensal microbiota may still be important for regulating the status of the colonic SILT, however, by a process different from the small intestine, involving IL-23 as an important (and colon-specific) factor for ILF development.

## ILF in Intestinal Inflammation

Despite the findings that ILF formation in the small intestine and the colon is initiated after birth and requires additional environmental factors, these structures may still be considered as SLO, since their development depends on the programed initial clustering of LTi cells into CP. In contrast, TLO can form as a consequence of an inflammatory process and are independent of the programed clustering of LTi cells. As the intestinal SILT already represents a flexible system of “inducible” SLO, the question remains whether true TLO do also develop in this organ during inflammation. Evidence that TLO induction, indeed, occurs in the intestine comes from studies using RORγt-deficient mice, which lack all RORγt-dependent ILC and, therefore, all programed peripheral lymphoid tissues, including LN, PP, and colonic patches. In these mice, numerous ILF-like B cell follicles containing germinal centers develop spontaneously in the colon, demonstrating that formation of TLO is possible in the intestine in the absence of LTi cells (54). It is likely that the spontaneous formation of such colonic TLO is compensating for the lack of other intestinal lymphoid tissues or critical components of the intestinal immune system (55). In fact, an increase in colonic ILF has also been observed in mice after the development of mLN, and PP was inhibited by the *in utero* blockage of LTβR and TNFRp55 or in mice that are deficient for the Ahr (43, 56). In RORγt-deficient mice, the development of colonic TLO was still dependent on LTβR signaling, and it was shown that B cells were the critical source of LTα_1_β_2_ in the absence of LTi cells (54). The induction of TLO in RORγt-deficient mice was strictly dependent on the microbiota and strongly enhanced upon induction of intestinal inflammation with dextran sodium sulfate (DSS). Interestingly, there was an intriguing correlation between the presence of TLO in the colon of RORγt-deficient mice and the severity of the colonic inflammation. The pathology could be reverted to levels seen in wild-type control mice by blocking the development of TLO using a LTβR–Ig fusion protein, suggesting that the TLO in this model indeed contribute to the severity of colitis. This finding complements data from other studies suggesting that increased formation of mucosal lymphoid tissues is associated with an exacerbation of intestinal pathology. In TNFΔARE mice, which serve as a TNFα-dependent model of Crohn’s Ileitis, induction of TLO within the chronically inflamed terminal ileum was observed (57). Aberrant production of CCL19 and CCL21 by these structures enhanced the density of infiltrating effector memory T cells and augmented their retention in the inflamed intestine, contributing to the perpetuation of ileitis. Also, the B cell follicles that develop in the colon of Ahr-deficient mice have been associated with intestinal pathology, such as the formation of anal prolapse, colonic hyperplasia, and increased inflammation upon intestinal infection (43, 58). There is ample evidence also from other studies in mice and in men that intestinal inflammation is associated with enhanced formation of structured lymphoid tissues (59–66) (summarized in Table 1). Yet, the contribution of these lymphoid organs to the perpetuation or exacerbation of the inflammatory pathology was, in the cases where it was tested, less evident.

**Table 1:** **Formation of lymphoid follicles in intestinal inflammation**.

Species	Phenotype	Reference
Ahr^−/−^ mice	Spontaneous lymphoid follicle formation in colon, anal prolapse, and increased intestinal inflammation upon infection	Fernandez-Salguero et al. (58) and Kiss et al. (43)
RORγt^−/−^ mice	Spontaneous lymphoid follicle formation in colon, increased follicle numbers and enhanced pathology during DSS colitis. Blocking of lymphoid follicle formation reduced pathology	Lochner et al. (54)
TNFΔARE mice	Lymphoid follicle induction in terminal ileum during spontaneous ileitis	McNamee et al. (57)
WT mice	Lymphoid follicle induction in the colon upon DSS colitis	Olivier et al. (66)
CD40L/B tg mice	Lymphoid follicle formation in small intestine and colon during spontaneous colitis	Kawamura et al. (65)
LN/PP-deficient mice	Formation of lymphoid follicles during DSS colitis, no correlation with disease severity	Spahn et al. (64)
Human	*De novo* formation of lymphoid follicle in UC and CD	Kaiserling (62)
Human	Description of lymphoid aggregates in UC lesions	Carlsen et al. (63)
Human	Description of lymphoid aggregates in CD lesions	Makiyama et al. (59), Surawicz and Belic (60), and Fujimura et al. (61)

*UC, ulcerative colitis; CD, Crohn’s disease*.

Of note, the question whether the lymphoid organs that form in intestinal inflammatory diseases also represent *de novo*-induced TLO instead of ILF that develop as SLO within the SILT network is, as of now, not completely clear. In this regard, the group of Reina Mebius recently reported the *de novo* formation of lymphoid tissues in the colon of mice treated with DSS (66). Although these structures showed all hallmarks of mature ILF in terms of the presence of B cell follicles, DC, FDC, and a vascular network, they also presented a distinct T cell area. It is likely that these lymphoid structures indeed represent *de novo*-formed TLO, since they were not observed in non-treated control mice. Thus, despite the lack of a marker allowing to definitely discriminate ILF from TLO, it may be possible to distinguish inflammation-induced TLO from ILF by structural differences, such as the presence of defined T cell areas. Interestingly, the same study also suggested a specific role for the nervous system in colonic TLO formation. It was shown that disrupting the innervation of the proximal colon by the vagus nerve prevented the induction of TLO upon DSS treatment, as a consequence of insufficient upregulation of CXCL13 and CCL20 expression by colonic stromal cells.

## ILF in Intestinal Infection

Under steady-state conditions, SILT functions mainly as a dynamic expandable system that contributes to the regulation of the intestinal commensal microbiota by the induction of IgA [reviewed in Ref. (38)]. It has been shown that ILF, as well as other gut lymphoid organs, harbor in their interior populations of specific commensal bacteria, such as *Alcaligenes* spp., *Achromobacter* spp., and others (67, 68). These lymphoid tissue-resident commensals could stimulate local ILC3 and T cell responses and induce the secretion of IL-10, which limited proinflammatory responses in the steady-state and protected mice during DSS-induced colitis (69). Besides their role in establishing mutualism between the host and the commensal microbiota, ILF may, however, also participate in the immune response against specific intestinal pathogens. In that respect, it has been shown that in mice lacking LNs and PP, but not ILF, *Salmonella typhimurium*-specific IgA responses could be induced upon oral infection with this pathogen (70). *Candidatus arthromicus* [also known as segmented filamentous bacteria (SFB)] has been described as a bacterial strain with marked immunostimulatory properties, although it does not cause overt immunopathology (71). SFB colonizes mainly the terminal ileum of mice, where it can directly attach to the epithelium and induce the maturation of several intestinal immune functions, including IgA production and Th17 induction (72, 73). In mice containing a normal set of lymphoid organs, PP represent a major site for SFB-induced IgA production (74). However, when mice were made devoid of LN, PP, as well as CP-derived ILF by LTβR–Ig treatment, SFB induced the formation of small intestinal TLO capable of initiating an IgA response to SFB. Interestingly, IgA-induction against a non-adherent and non-virulent strain of *Escherichia coli* occurred in PP, but was completely abolished in mice lacking PP. Overall, these data suggest that specific bacteria with immunostimulatory or pathogenic properties can stimulate the formation of ILF, which can substitute for PP as inductive sites for intestinal IgA. Interestingly, pathogen-induced formation of lymphoid follicles has also been observed in the mucosal tissue of the stomach. Although the stomach generally lacks organized or diffused lymphoid tissues, infection with *Helicobacter pylori* can induce the development of gastric lymphoid follicles in mice, presumably in an LTi cell-dependent manner (75). Of note, also intestinal parasites, such as the nematode *Trichuris muris* can induce the formation of ILFs in the colon of infected mice (76).

Evidence for a functional role of ILFs in host protection against intestinal infection comes from studies using the bacterium *Citrobacter rodentium*, a non-invasive Gram-negative mouse enteric pathogen that forms attaching and effacing (A/E) lesions in the intestinal epithelium, modeling the infection process of the human pathogens, enterohemorrhagic *E. coli* (EHEC) and enteropathogenic *E. coli* (EPEC) (77). During the early phase of infection, RORγt^+^ ILC3 produces large amounts of IL-22 (78–80), which has a major protective role, e.g., by inducing the expression of antimicrobial proteins, such as RegIIIβ and RegIIIγ, by intestinal epithelial cells (81). Mice that lack ILC3 are highly susceptible to intestinal infection with *C. rodentium* (78, 80), largely recapitulating the phenotype of IL-22-deficient mice (82). Importantly, the LTβR-pathway also plays an essential role for the defense against this pathogen, since LTβR-deficient mice were found to be highly susceptible to *C. rodentium* and to succumb rapidly upon infection (83, 84). It turned out that RORγt^+^ ILC were the critical cell type for the activation of LTβR-signaling *via* their surface expression of LTα_1_β_2_ (84–86). It was furthermore demonstrated that the LT pathway was required for the subsequent induction of IL-22 production by ILC. Intriguingly, IL-22^+^ ILC closely interacted with DCs in colonic ILF, and it was shown that LTβR-expression by CD11c^+^ DC is a prerequisite for IL-22 induction in ILC (86). The data provided by the study of Tumanov and colleagues suggest that ILF, indeed, provide the necessary microenvironment for the close interaction between RORγt^+^ ILC and DC. The LTα_1_β_2_–LTβR-mediated interaction results in IL-23 secretion by the DC, which in turn activates IL-22 production from the ILC. Importantly, both disruption of the LTβR pathway and IL-22 deficiency interfered with the induction of ILF and impaired their structural integrity (85). However, the surprising role of IL-22 for ILF formation was only observed after infection with *C. rodentium*, but not during the steady state. Together, these findings indicate that ILF not only provide the critical environment for the induction of an effective immune response toward intestinal infection but also demonstrate an important role for RORγt^+^ ILC-derived IL-22 for the induction and maintenance of ILF under infectious conditions in the gut. Notably, a critical role of IL-22 has also been reported recently in a model of adenovirus-induced development of TLO in the salivary glands of mice (87). In the absence of IL-22, the recruitment of B cells into the developing TLO structures was significantly impaired, resulting in a strong reduction of autoantibody formation. It was shown that IL-22 production was necessary for the upregulation of CXCL12 and CXCL13 from epithelial and stromal cells within TLO, and therapeutic blocking of IL-22 inhibited the expression of both chemokines and the correct assembly of TLO. Although ILC were identified as IL-22 producers, the majority of cells expressing this cytokine at the early phase were γδ T cells and αβ T cells at the later stage. These findings together, thus, suggest important regulatory role for IL-22 in the formation of lymphoid organs under inflammatory or infectious conditions at mucosal sites.

## Concluding Remarks

In contrast to most other lymphoid tissues of the body, the formation of ILF in the gut is initiated after birth and unfolds upon the reception of environmental signals associated with the uptake of food-derived compounds and microbial colonization. Despite these differences, ILF can be considered as SLO, since their development strictly requires the LTi-dependent formation of CP. This distinguishes ILF from TLO, which can be induced also in the absence of LTi cells and in response to an inflammatory trigger. Recent experiments in mice suggest that inflammation not only leads to an expansion of the existing SILT network but also to the induction of TLO in the gut. Although it is not clear so far, to what extent TLO contribute to the inflammatory process and pathology in intestinal inflammation, disrupting TLO formation may represent a strategy for the treatment of chronic inflammatory disorders. Regarding its prominent role in lymphoid neogenesis, targeting the LTβR pathway may prove effective in blocking TLO development during inflammation. However, the human phase II studies that analyzed therapeutics targeting the lymphotoxin pathway showed only very limited effects in improving the symptoms of primary Sjögren’s syndrome or rheumatoid arthritis (88, 89). Another promising approach to interfere with TLO development is the blockade or neutralization of CXCL13 in order to prevent B cell recruitment into lymphoid structures. In this regard, antibody-mediated neutralization of CXCL13 in a mouse model for Sjögren’s syndrome resulted in improved disease phenotype and reduced lymphoid follicles in the submandibular glands (90). CXCL13 neutralization had also a beneficial effect in mouse models for rheumatoid arthritis and multiple sclerosis (91), although the effect on TLO formation was not determined in this study. Blocking CXCL13 in the NOD-mouse model for diabetes disrupted the organization of TLO in the pancreas of the mice, however, without affecting disease severity (92). These findings indicate that the effect of anti-CXCL13 treatment may be influenced by the specific characteristics of the inflammatory model. In that respect, it will be interesting to see whether blocking of CXCL13 also impacts on gut inflammation and TLO development in the intestine.

Besides the well-known factors that are required for the development of ILF, there are also cytokines like IL-22 entering the stage that may have a specific function in ILF biology. Also, IL-23, besides its established proinflammatory role within the T_H_17–IL-23 axis (93), seems to influence ILF formation specifically in the colon, and has been associated with TLO formation in rheumatoid arthritis (94). Also, IL-17, which together with IL-22 is produced by neonatal RORγt^+^ LTi cells as well as by RORγt-expressing T cell subsets, has been implicated in TLO formation in the lung (95) and in a model of experimental allergic encephalomyelitis (96). Nevertheless, the significance of these cytokines for the development and structural maintenance of organized lymphoid tissues may strongly depend on the inflammatory context and the target organ. While, so far, no role for IL-17 in ILF formation has been reported, its requirement for TLO formation in the lung also depends strongly on the model used (97).

Increasing our knowledge on the similarities, but in particular also the differences, in the mechanisms that govern postnatal development of lymphoid tissues such as ILF in the small intestine and the colon may offer novel strategies for immunomodulation. It should also be taken into account that targeting cytokines, e.g. within the T_H_17–IL-23 axis, for the treatment of inflammatory disorders may in addition to the direct anti-inflammatory effects also critically influence the formation and function of lymphoid tissues.

## Author Contributions

MB and ML contributed equally to designing and writing of the review.

## Conflict of Interest Statement

The authors declare that the research was conducted in the absence of any commercial or financial relationships that could be construed as a potential conflict of interest.

## Abbreviations

CP, cryptopatches; ILC, innate lymphoid cells; ILF, isolated lymphoid follicles; LTi, lymphoid tissue inducer; LTo, lymphoid tissue organizer; PP, Peyer’s patches; SILT, solitary intestinal lymphoid tissues; SLO, secondary lymphoid organs; TLO, tertiary lymphoid organs.
